# Network Pharmacology-Based Approach to Comparatively Predict the Active Ingredients and Molecular Targets of Compound Xueshuantong Capsule and Hexuemingmu Tablet in the Treatment of Proliferative Diabetic Retinopathy

**DOI:** 10.1155/2021/6642600

**Published:** 2021-03-05

**Authors:** Hongyan Yao, Danli Xin, Zongyi Zhan, Zijing Li

**Affiliations:** ^1^Ningbo Eye Hospital, Ningbo University, Ningbo 315000, China; ^2^Ningbo Key Laboratory of Ocular Tissue Transplantation, Ningbo 315000, China; ^3^Department of Ophthalmology, Sun Yat-Sen Memorial Hospital, Sun Yat-Sen University, Guangzhou 510020, China; ^4^Guangdong Provincial Key Laboratory of Malignant Tumor Epigenetics and Gene Regulation, Sun Yat-Sen Memorial Hospital, Sun Yat-Sen University, Guangzhou 510020, China

## Abstract

**Background:**

Compound Xueshuantong capsule (CXC) and Hexuemingmu tablet (HXMMT) are two important Chinese patent medicines (CPMs) frequently used to treat proliferative diabetic retinopathy (PDR), especially when complicated with vitreous hemorrhage (VH). However, a network pharmacology approach to understand the therapeutic mechanisms of these two CPMs in PDR has not been applied.

**Objective:**

To identify differences in the active ingredients between CXC and HXMMT and to comparatively predict and further analyze the molecular targets shared by these CPMs and PDR. *Materials and methods*. The differentially expressed messenger RNAs (mRNAs) between normal retinal tissues in healthy individuals and active fibrovascular membranes in PDR patients were retrieved from the Gene Expression Omnibus database. The active ingredients of CXC and HXMMT and the targets of these ingredients were retrieved from the Traditional Chinese Medicine Systems Pharmacology database. The intersections of the CPM (CXC and HXMMT) targets and PDR targets were determined. Then, Gene Ontology and Kyoto Encyclopedia of Genes and Genomes (KEGG) analyses were performed, and the ingredient-target networks, protein-protein interaction networks, and KEGG-target (KEGG-T) networks were constructed.

**Results:**

CXC contains 4 herbs, and HXMMT contains 19. *Radix salviae* is the only herb common to both. CXC had 34 potential therapeutic targets in PDR, while HXMMT had these 34 and 10 additional targets. Both CPMs shared the following main processes: response to reactive oxygen species and oxidative stress, regulation of blood vessel diameter and size, vasoconstriction, smooth muscle contraction, hemostasis, and blood coagulation. The shared pathways included the AGE-RAGE signaling pathway in diabetic complications, TNF signaling pathway, relaxin signaling pathway, and IL-17 signaling pathway.

**Conclusions:**

Both CXC and HXMMT include components effective at treating PDR and affect the following main processes: response to reactive oxygen species and oxidative stress, regulation of blood vessels, and blood coagulation. *Radix salviae*, the only herb common to both CPMs, contains many useful active ingredients. The PDR-CXC and PDR-HXMMT networks shared 34 common genes (RELA, HSPA8, HSP90AA, HSP90AB1, BRCA, EWSR1, CUL7, HNRNPU, MYC, CTNNB1, MDM2, YWHAZ, CDK2, AR, FN1, HUWE1, TP53, TUBB, EP300, GRB2, VCP, MCM2, EEF1A1, NTRK1, TRAF6, EGFR, PRKDC, SRC, HDAC5, APP, ESR1, AKT1, UBC, and COPS5), and the PDR-HXMMT network has 10 additional genes (RNF2, VNL, RPS27, COPS5, XPO1, PARP1, RACK1, YWHAB, and ITGA4). The top 5 pathways with the highest gene ratio in both networks were the AGE-RAGE signaling pathway in diabetic complications, TNF signaling pathway, relaxin signaling pathway, IL-17 signaling pathway, and focal adhesion. Additional pathways such as neuroactive ligand-receptor interaction, chemokine signaling pathway, and AMPK signaling pathway were enriched with HXMMT targets. Thus, HXMMT has more therapeutic targets shared by different active ingredients and more abundant gene functions than CXC, which may be two major reasons why HXMMT is more strongly recommended than CXC as an auxiliary treatment for new-onset VH secondary to PDR. However, the underlying mechanisms still need to be further explored.

## 1. Background

Diabetic retinopathy (DR), a serious complication of diabetes mellitus (DM) caused by microvascular ischemia and hypoxemia, affects approximately 35% of DM patients and an estimated > 90 million people worldwide [1, 2]. The prevalence of proliferative diabetic retinopathy (PDR), a vision-threatening type of DR characterized by retinal neovascular and even vitreous hemorrhage (VH), is nearly 7% [3]. PDR dramatically decreases patients' quality of life and contributes to a massive economic burden. Therefore, it is crucial to develop effective pharmaceutical preparations to treat PDR based on its pathological mechanisms.

For thousands of years, traditional Chinese medicines (TCMs) have been used by Chinese people to treat DM and its complications [4]. Compound Xueshuantong capsule (CXC) and Hexuemingmu tablet (HXMMT) are two important Chinese patent medicines (CPMs) that are frequently used to treat PDR, especially when complicated with VH [5, 6]. According to observations in daily clinical practice and the results of some clinical studies, CXC and HXMMT are crucial auxiliary treatments to eliminate VH secondary to PDR as well as to improve retinal hemodynamics in PDR [6]. Correspondingly, previous experimental studies have shown that CXC and HXMMT may exert protective effects on retinal capillary endothelial cells and nerve cells by regulating multiple pathways [7–9]. However, most CMPs are composed of different types of herbs, and every herb is further composed of multiple active ingredients. Thus, a single CPM may target numerous PDR-related molecules, and the pharmacological mechanisms are complex. Moreover, in clinical practice, HXMMT more effectively eliminates new-onset VH secondary to PDR than HXMMT and is more frequently recommended by TCM doctors for new-onset VH treatment, but its potential mechanisms are poorly understood. Thus, further research is needed to better understand the underlying regulatory and interactive mechanisms of different active ingredients in CXC and HXMMT.

Network pharmacology analysis is a convenient and systematic approach to identify core targets shared by drugs and diseases [10]. It can also be performed to identify potential pathways for disease interventions, providing insight into the complex mechanisms of Chinese herbal formulas used to treat diseases. The aim of our study was to find differences in active ingredients between CXC and HXMMT and to comparatively predict and further analyze the molecular targets shared by these drugs and PDR.

## 2. Materials and Methods

**Figure 1:**
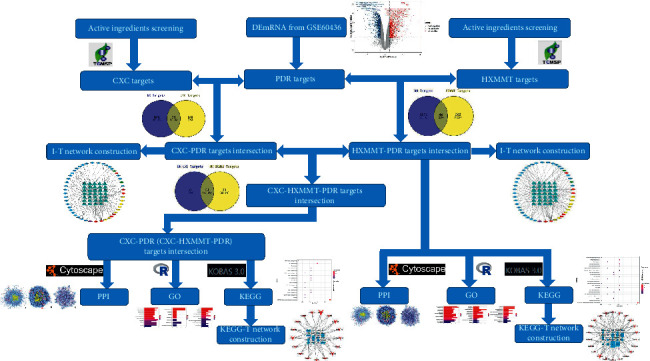
Flowchart of the complete analysis procedure. DEmRNA, differentially expressed messenger RNA; CXC, compound Xueshuantong capsule; PDR, proliferative diabetic retinopathy; HXMMT, Hexuemingmu tablet; I-T, ingredient-target; PPI, protein-protein interaction; GO, Gene Ontology; KEGG, Kyoto Encyclopedia of Genes and Genomes; KEGG-T, Kyoto Encyclopedia of Genes and Genomes-target.

The general procedure was as follows (described in detail in Section 2.1). First, differentially expressed mRNAs (DEmRNAs) between normal retinal tissues in healthy individuals and abnormal retinal membranes in PDR patients were acquired from the database. The chosen DEmRNAs according to certain criteria were defined as PDR targets (described in detail in Section 2.2). Second, active ingredient screening was, respectively, performed in CXC and HXMMT; thus, the CXC and HXMMT targets were obtained (described in detail in Sections 2.3 and 2.4). Third, the intersections between CXC and PDR targets (CXC-PDR targets) as well as between HXMMT and PDR targets (HXMMT-PDR targets) were determined. Afterward, intersections between CXC-PDR targets and HXMMT-PDR targets were identified. We found that all the CXC-PDR targets were completely included in the HXMMT-PDR targets. Finally, the ingredient-target (I-T) networks, protein-protein interaction (PPI) networks, Kyoto Encyclopedia of Genes and Genomes-target (KEGG-T) networks, and Gene Ontology (GO) analyses were performed for CXC-PDR and HXMMT-PDR targets. A flowchart of the procedure is shown in Figure 1, which provides a detailed description of each step.

### 2.1. Messenger RNA (mRNA) Data Collection and Differential Expression Analysis

The microarray data used in this study were retrieved from the Gene Expression Omnibus (GEO) database (http://www.ncbi.nlm.nih.gov/gds/). The mRNA expression data were acquired from dataset GSE60436, which contains 3 samples from normal retinal tissues and 3 from active fibrovascular membranes in PDR patients. The raw expression data were first normalized, and analysis of DEmRNAs was then performed using the limma package based on the R language. The criteria for the selection of DEmRNAs were an adjusted *P* value of <0.05 and a |log2FC| value of >1, and the selected DEmRNAs were defined as PDR targets.

### 2.2. Active Ingredient Screening

A total of 499 Chinese herbs and 12144 chemical ingredients from the Chinese pharmacopoeia (2010) were registered in the Traditional Chinese Medicine Systems Pharmacology (TCMSP) database (https://tcmspw.com/index.php), a platform that provides pharmacokinetic characteristics and targets of each ingredient in these herbs. Oral bioavailability (OB) and drug-likeness (DL) are two parameters commonly used to screen active ingredients. The OB is the percentage of the orally administered dose of the unchanged drug that enters the systemic blood circulation, and it is an important pharmacokinetic indicator. DL is used to assess whether the ingredients function as known drugs.

Each herb contained in CXC and HXMMT was searched in the TCMSP database, and all ingredients were obtained. According to most traditional Chinese herbs studies [10–12], an ingredient with an OB of ≥30 and a DL of ≥0.18 was considered an active ingredient, and the targets of these ingredients (CXC and HXMMT targets) were retrieved from the database.

### 2.3. Network Construction

Cytoscape 3.6.1 (http://cytoscape.org/) was used to generate all visual network diagrams, including the I-T, PPI, and KEGG-T networks. The intersections of the formulas' targets (CXC and HXMMT targets) and the PDR targets were determined (CXC-PDR and HXMMT-PDR targets). Accordingly, two I-T networks were constructed. PPI networks were constructed using the Bisogenet 3.0.0 plugin in Cytoscape 3.6.1 based on the following databases: the Database of Interacting Proteins, the Biological General Repository for Interaction Datasets, the Human Protein Reference Database, the IntAct Molecular Interaction Database, the Molecular INTeraction Database and the Binding Database. The CytoNCA plugin was used to perform topological analyses. Degree centrality and betweenness centrality were the measures selected to represent the topological features of each node in the network. Degree centrality represents the number of edges linked by a node, while betweenness centrality represents the proximity of a node to other nodes. KEGG-T networks were constructed after enrichment analyses were performed.

### 2.4. Functional Enrichment Analysis

GO analyses were performed using the clusterProfiler package based on the R language. The criterion for the selection of GO processes was a *P* value of <0.05. KEGG pathway analyses were performed using an online biological tool, KEGG Orthology Based Annotation System 3.0 (KOBAS 3.0, http://kobas.cbi.pku.edu.cn). The criteria for the selection of KEGG pathways were a *P* value of <0.01 and a gene count of ≥3. Visualizations were performed using the ggplot2 R package.

## 3. Results

### 3.1. Identification of PDR Targets

**Figure 2:**
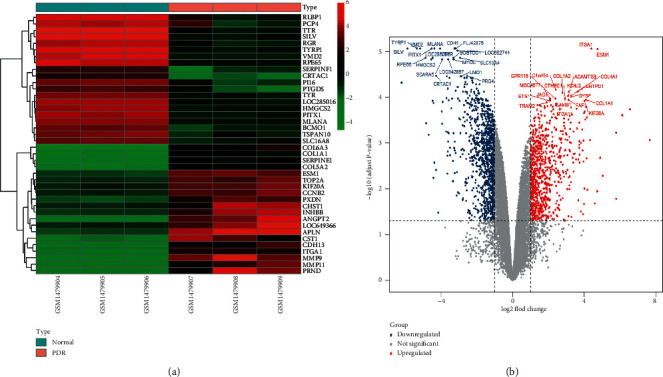
Heatmap (a) and volcano plot (b) of DEmRNAs (PDR targets) in microarray dataset GSE60436. The top 20 downregulated and upregulated DEmRNAs are shown in the heatmap and the volcano plot.

Analysis of the microarray dataset GSE60436 showed that 1915 mRNAs (819 upregulated mRNAs and 1096 downregulated mRNAs) were differentially expressed in PDR patients compared with individuals without PDR (Figure 2).

### 3.2. I-T Network Construction

**Figure 3:**
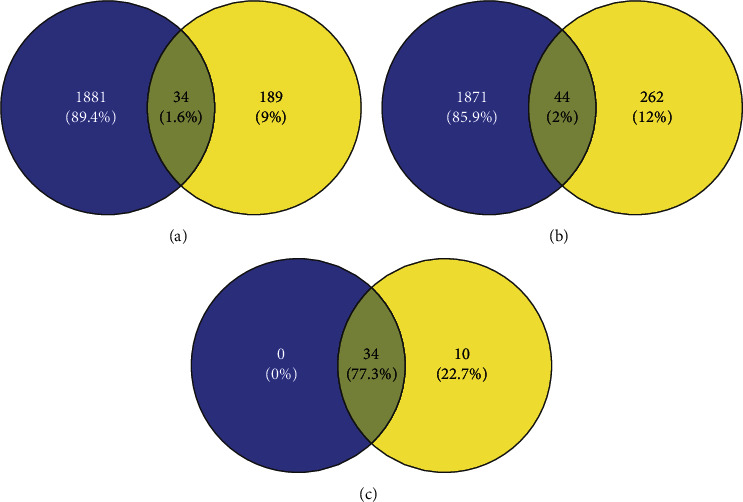
(a) Intersection of compound Xueshuantong capsule (CXC) targets (blue) and proliferative diabetic retinopathy (PDR) targets (yellow). (b) Intersection of Hexuemingmu tablet (HXMMT) targets (blue) and PDR targets (yellow). (c) Intersection of PDR-CXC targets (blue) and PDR-HXMMT targets (yellow).

**Figure 4:**
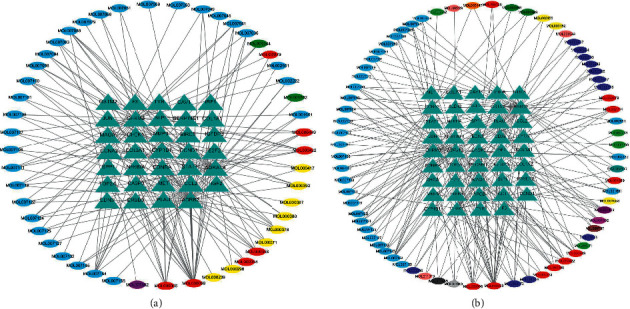
Ingredient-target networks of different Chinese patent medicines. The triangles and circles represent potential therapeutic targets and active ingredients, respectively. (a) Network for the compound Xueshuantong capsule. The blue circles represent active ingredients in *Radix salviae*, the green circles represent active ingredients in *Panax notoginseng*, the yellow circles represent active ingredients in *Hedysarum multijugum* Maxim., the purple circles represent active ingredients in *Figwort root*, and the red circles represent active ingredients in multiple herbs. (b) Network for the Hexuemingmu tablet. The light blue circles represent active ingredients in *Radix salviae*, the bluish green circles represent active ingredients in *Chuanxiong rhizoma*, the light purple circles represent active ingredients in *Radix paeoniae rubra*, the yellow circles represent active ingredients in *Gentianae radix et rhizoma*, the dark blue circles represent active ingredients in *Scutellariae radix*, the light green circles represent active ingredients in *Cassiae semen*, the pink circles represent active ingredients in *Chrysanthemi flos*, the dark green circles represent active ingredients in *Ecliptae herba*, the light gray circles represent active ingredients in *Equiseti hiemalis herba*, the orange circles represent active ingredients in *Fructus ligustri lucidi*, the dark purple circles represent active ingredients in *Pollen typhae*, the dark gray circles represent active ingredients in *Crataegus pinnatifida* Bunge, the brown circles represent active ingredients in *Prunellae spica*, and the red circles represent active ingredients in multiple herbs.

CXC contains 4 herbs, and HXMMT contains 19 herbs (Table 1). *Radix salviae* is the only herb common to both CPMs. Through screening the active ingredients, we found 34 potential therapeutic targets in PDR for CXC (Figure 3(a)) and the same 34 and 10 additional therapeutic targets in PDR for HXMMT (Figure 3(b)). The ten additional targets of HXMMT were CYCS, APOD, GOT1, PECAM1, ALDH2, COL1A2, CD300A, PTGER2, CHGA, and CD36. Accordingly, the active ingredients and the potential therapeutic targets were used to construct I-T networks for CXC (Figure 4(a)) and HXMMT (Figure 4(b)). Then, the intersections of the PDR-CXC targets and the PDR-HXMMT targets were determined, and the complete set of potential therapeutic targets of CXC was found to be included among the targets of HXMMT (Figure 3(c)).

**Figure 5:**
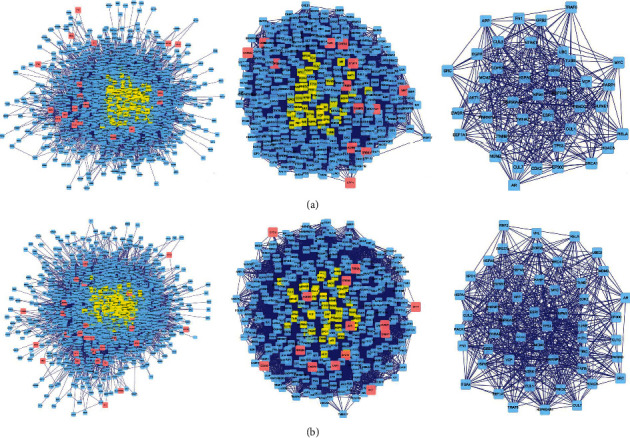
Topological screening process of the protein-protein interaction (PPI) networks. Left: original PPI network; middle: PPI network after the first screen; right: PPI network after the second screen. (a) Screening process of the compound Xueshuantong capsule. (b) Screening process of the Hexuemingmu tablet.

A PPI network containing 1825 nodes and 36349 edges was constructed for the set of PDR-CXC targets, while another PPI network containing 2004 nodes and 38863 edges was constructed for the set of PDR-HXMMT targets. The screening parameters were degree centrality and betweenness centrality. The thresholds were set at a degree centrality of ≥61 in the first screen and a betweenness centrality of ≥600 in the second screen. After the first screen, 350 nodes and 10843 edges were included in the PDR-CXC network, while 386 nodes and 11975 edges were included in the PDR-HXMMT network. After the second screen, 39 nodes and 406 edges were included in the PDR-CXC network, while 51 nodes and 628 edges were included in the PDR-HXMMT network. All the key genes in the PDR-CXC network were also included in the PDR-HXMMT network (RELA, HSPA8, HSP90AA, HSP90AB1, BRCA, EWSR1, CUL7, HNRNPU, MYC, CTNNB1, MDM2, YWHAZ, CDK2, AR, FN1, HUWE1, TP53, TUBB, EP300, GRB2, VCP, MCM2, EEF1A1, NTRK1, TRAF6, EGFR, PRKDC, SRC, HDAC5, APP, ESR1, AKT1, UBC, and COPS5). In addition, RNF2, VNL, RPS27, COPS5, XPO1, PARP1, RACK1, YWHAB, and ITGA4 were included only in the PDR-HXMMT network. The topological screening processes of the PPI networks are shown in Figure 5.

### 3.3. GO and KEGG Pathway Analyses

**Figure 6:**
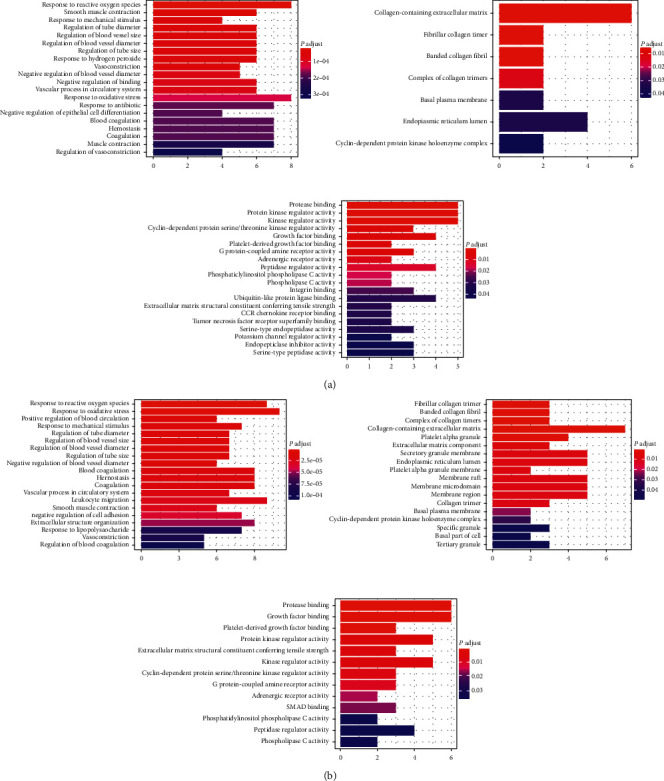
Gene Ontology (GO) analysis results. Left: biological process; middle: cellular component; right: molecular function. (a) GO terms enriched with targets of the compound Xueshuantong capsule. (b) GO terms enriched with targets of the Hexuemingmu tablet.

The top 20 or all GO processes (when the number of processes was smaller than 20) in the biological process (BP), cellular component (CC), and molecular function (MF) categories were identified (Figure 6). Both CXC and HXMMT may have therapeutic effects on PDR mainly via the following processes in the BP category: response to reactive oxygen species and oxidative stress, regulation of blood vessel diameter and size, vasoconstriction, smooth muscle contraction, hemostasis, and blood coagulation. Additionally, GO terms, such as positive regulation of blood circulation, negative regulation of cell adhesion, extracellular structure organization, and response to lipopolysaccharide, were enriched in HXMMT targets. In the CC category, the therapeutic targets of both CXC and HXMMT were associated mainly with the terms collagen-containing extracellular matrix, fibrillar collagen trimer, and banded collagen fibril. Moreover, HXMMT targets were enriched in the term platelet alpha granule. In addition, CXC and HXMMT targets overlapped in GO MF terms, most of which were related to protein and enzyme binding and regulation.

**Figure 7:**
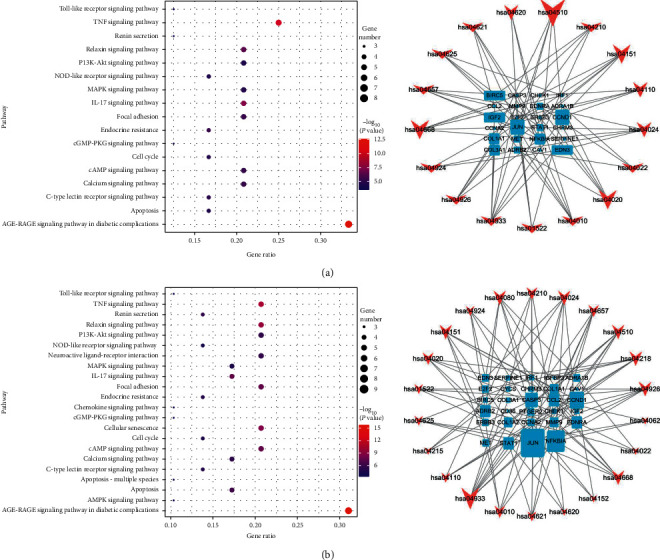
Kyoto Encyclopedia of Genes and Genomes (KEGG) pathway analysis results (left) and KEGG-target networks (right). In the right panel, the arrows and squares represent pathways and potential therapeutic targets, respectively. The size of the arrows represents the ratio of the corresponding therapeutic targets, while the size of the therapeutic targets represents the ratio of the corresponding pathways. (a) Pathways enriched with targets of the compound Xueshuantong capsule. (b) Pathways enriched with targets of the Hexuemingmu tablet.

**Figure 8:**
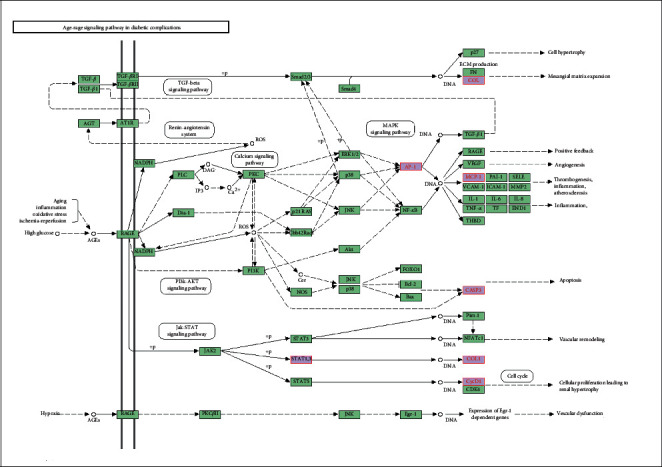
Most significant pathway and key genes (marked in red) among the proliferative diabetic retinopathy treatment targets of the compound Xueshuantong capsule and Hexuemingmu tablet.

After KEGG pathway analyses were performed using KOBAS 3.0, possible PDR-related pathways were selected, and visualizations were generated using the ggplot2 R package. All genes and pathways enriched with CXC targets were also enriched with HXMMT targets, and HXMMT had 5 additional target genes: IGFBP3, CYCS, CD36, PTGER2, and COL1A2. The pathway that was the most significantly enriched and had the highest gene ratio in both analyses was the AGE-RAGE signaling pathway in diabetic complications. Other common pathways included the TNF signaling pathway, relaxin signaling pathway, IL-17 signaling pathway, and focal adhesion. Additional pathways, such as neuroactive ligand-receptor interaction, chemokine signaling pathway, and AMPK signaling pathway, were enriched with HXMMT targets. Then, the enriched pathways and their related target genes were used to construct KEGG-T networks for CXC and HXMMT. These results are shown in Figure 7. The most significant pathway and key genes among the PDR treatment targets of CXC and HXMMT are shown in Figure 8.

## 4. Discussion

The GO terms enriched with CXC and HXMMT targets were similar and focused mainly on the response to reactive oxygen species and oxidative stress, regulation of blood vessels, and blood coagulation.

*Radix salviae* is an important component that is closely related to the response to reactive oxygen species and oxidative stress. In an in vitro model of hypoxia and reoxygenation, Hu's [13] study showed that *Radix salviae* obviously alleviated cardiomyocyte apoptosis and protected mitochondrial function and cell membrane skeleton integrity in H9c2 cells. In addition, Zhang's experiment in rodents [14] revealed that the Danshen *(Radix salviae*) dripping pill inhibited apoptosis and exerted neuroprotective effects in the retinas of diabetic rats by increasing the expression of Bcl-2, Bcl-2-associated X, and caspase-3 in diabetic rats. Moreover, according to the I-T networks of CXC and HXMMT, *Radix salviae* contained a greater number of active ingredients related to potential therapeutic targets in PDR compared with the other herbal components, suggesting that *Radix salviae* may play a crucial role in PDR treatment. A randomized controlled trial (RCT) performed by Lian and colleagues [5] showed that a *Radix salviae*-containing Chinese herbal product was effective in treating DR and in delaying the progression from non-PDR to PDR by reducing the area of capillary nonperfusion and degree of vascular leakage. Our study identified tanshinone as one of the most important active ingredients of *Radix salviae*. According to previous studies, tanshinone exerts protective effects on retinal pigment epithelium and retinal endothelial cells [15–17].

Regarding circulatory-related effects, CXC is considered to be an effective complementary medicine to treat ischemic vascular diseases, such as cerebral infarction and cardiovascular diseases [18, 19]. Lyu's study [18] showed that CXC combined with conventional treatments had better clinical effects than conventional treatments alone. Moreover, a significant reduction in the IL-6 and hs-CRP levels was noticed when CXC was combined with conventional treatments. Regarding CXC and DR, some studies [20, 21] have shown that CXC contributes to the attenuation of streptozotocin- (STZ-) induced retinal lesions, including the amelioration of increases in erythrocyte aggregation, plasma viscosity, and acellular vessel and pericyte loss, by reversing the hyperexpression of vascular endothelial growth factor (VEGF) and intercellular adhesion molecule-1 (ICAM-1) and endothelin-1 (ET-1) and the hypoexpression of pigment epithelium-derived factor (PEDF) and occludin in the retinas of STZ-induced rats. In addition, Liu's study [22] showed that different core bioactive ingredients in CXC had novel therapeutic uses in managing blood circulation. Panaxytriol and ginsenoside Rb1 were related to red blood cell aggregation, while angoroside C was involved in platelet aggregation. Protocatechualdehyde was related to intrinsic clotting activity, while calycosin-7-O-beta-D-glucoside was related to extrinsic clotting activity. In Sun et al.'s study [23], the systolic and diastolic velocity decreased while the resistance and pulsatility index increased in diabetic rat retinas. Furthermore, they also proved that the protective effects of DR were mediated by coagulation cascades and the peroxisome proliferator-activated receptor (PPAR) signaling pathway. Xing et al.'s study [6] showed that CXC mainly affected blood vessels by protecting high glucose-injured retinal vascular endothelial cells via YAP-mediated effects. However, the effect of HXMMT has seldom been evaluated in DR. Indeed, the only study was conducted by Long et al. [7] in rat models of branch retinal vein occlusion (BRVO), which indicated that HXMMT may alleviate retinal edema by regulating the expression of VEGF-*α* and improving microcirculation. Further studies should be performed to clarify the mechanism of HXMMT.

Utilizing a network pharmacology approach, Piao et al. [11] found that MMP9 and IGF-1 (an IGF family member contained in *Radix salviae*) may be key therapeutic targets in DR. Consistent with Piao's result, we found that MMP9 was included in both the CXC and HXMMT I-T networks. Matrix metalloproteinases (MMPs) play an important role in the migration, differentiation, and proliferation of cells [24]. Hyperglycemia may increase the activity of MMP9 and, therefore, provides growth space and nutrients for neovascularization by degrading the basement membrane and relaxing the cell structure [25]. A previous study [26] suggested that MMP9 was upregulated in the DM heart and that knockout of MMP9 in the DM was cardioprotective. Activation of MMPs (MMP-2 and MMP-9) in the retina is an early event in DR. Therefore, activated MMPs increased retinal capillary cell apoptosis and mitochondrial damage [27]. In addition, IGF-2, another IGF family member, was included in our networks but is not targeted by *Radix salviae*. IGF is expressed in many tissues, including the retina, where it is found in cells such as retinal endothelial cells and retinal pigment epithelial cells. IGF is a crucial regulator of cell differentiation and is closely related to blood-retinal barrier breakdown and retinal neovascularization [28, 29]. However, IGF-2 was related to 2 herbal components of CXC (*Huangqi and Sanqi*) and 9 herbal components of HXMMT (*Cheqianzi, Chishao, Huangqin, Mohanlian, Mudanpi, Muzei, Nvzhenzi, Puhuang*, *and Xiakucao*), implying that different active ingredients may share common therapeutic targets. Combined and stronger therapeutic effects may be exerted on a therapeutic target shared by a greater number of active ingredients. HXMMT contains more components than CXC; therefore, HXMMT may have more therapeutic targets. In addition, many of the genes targeted only by HXMMT but not by CXC were related to circulation and blood coagulation. For instance, CYCS was shown to be involved in blood platelet formation and regulatory processes [30, 31]. APOD, a crucial component of lipoproteins that transports lipids and stabilizes the structure of lipoproteins, was found to also be closely related to angiogenesis, a critical pathophysiological process in PDR [32]. PECAM1 was suggested to play an important role in the maintenance of human vascular endothelial barrier integrity and function [33]. Similarly, our topological analysis showed that some genes targeted only by HXMMT had many other functions. For example, YWHAB may perform specific functions in rod photoreceptors [34]. RACK1 may promote the expression of VEGF in endothelial cells and subsequently facilitate angiogenesis [35]. PARP1, activated by reactive oxygen species, was proven to be involved in inflammation, cell death, and retinal disease progression [36, 37]. In summary, its stronger effects at a given dose and more numerous gene targets may be two major reasons why HXMMT is more strongly recommended than CXC by TCM doctors for treating fresh VH secondary to PDR.

Similar to Li et al.'s research [38], the AGE-RAGE signaling pathway and TNF signal pathway were enriched in CXC in our study. HXMMT and CXC shared many pathways in our study, and the AGE-RAGE signaling pathway in diabetic complications was the most significantly enriched pathway. RAGE is expressed in almost all retinal cells. Retinal Müller cells, the major glial cells in the retina, play a critical role in maintaining the structure and normal functions of the retina, and these cells express high levels of RAGE [39]. In addition, Zong et al.'s study [40] demonstrated that RAGE plays an essential role in retinal neurodegeneration induced by diabetes and that early induction of RAGE expression by hyperglycemia in retinal Müller cells contributes to the increased levels of proinflammatory cytokines, including VEGF (a crucial downstream growth factor in angiogenesis) and monocyte chemoattractant protein-1 (MCP-1), both in vivo and in vitro. Moreover, Hirata et al. [41] found that increased production of VEGF secondary to retinal Müller cell activation may account for neovascularization in PDR. Therefore, the AGE-RAGE signaling pathway may not only provide neuroprotection in DR but also participate in crosstalk between neuroprotection and vascular protection. The difference in the enriched genes between CXC and HXMMT was that one additional gene (COL1A2) was included among the HXMMT targets. COL1A2 has seldom been studied in DR; Zou et al.'s research [42] is the only DR study involving COL1A2 to date. Zou revealed that silencing of circular RNA COL1A2 (circCOL1A2) suppresses angiogenesis during PDR progression by regulating the miR-29b/VEGF axis, suggesting that circCOL1A2 and its related genes may be therapeutic targets in DR.

According to the pathway map, the AGE-RAGE signaling pathway in diabetic complications is also closely associated with the PI3K-Akt signaling pathway and VEGF. The PI3K-Akt signaling pathway is one of the most frequently studied pathways in DR [43–45]. The proliferation, migration, and invasion of retinal vascular endothelial cells, retinal pericytes, retinal pigment epithelial cells, and microglial cells can be regulated through this pathway [46–49]. A series of pathophysiological processes, including oxidative stress regulation, inflammatory response regulation, angiogenesis, and neuroprotective regulation, are also involved. Another common and well-known pathway, the TNF signaling pathway, is closely related to inflammation, which is a crucial process in DR progression [50, 51]. Gao's study [51] revealed that hypoxia inducible factor subtype 1*α* in diabetic retina is likely to play a role in dysfunction and vulnerability related to DR progression via TNF-*α*.

The genes and pathways mentioned above and whether CXC/HXMMT regulates their functions are summarized in Table 2.

## 5. Conclusions

Both CXC and HXMMT include components effective in treating PDR and affect the following main processes: response to reactive oxygen species and oxidative stress, regulation of blood vessels, and blood coagulation. *Radix salviae*, the only herb common to both CPMs, contains many useful active ingredients. The PDR-CXC and PDR-HXMMT networks shared 34 common genes (RELA, HSPA8, HSP90AA, HSP90AB1, BRCA, EWSR1, CUL7, HNRNPU, MYC, CTNNB1, MDM2, YWHAZ, CDK2, AR, FN1, HUWE1, TP53, TUBB, EP300, GRB2, VCP, MCM2, EEF1A1, NTRK1, TRAF6, EGFR, PRKDC, SRC, HDAC5, APP, ESR1, AKT1, UBC, and COPS5), and the PDR-HXMMT network has 10 additional genes (RNF2, VNL, RPS27, COPS5, XPO1, PARP1, RACK1, YWHAB, and ITGA4). The top 5 pathways with the highest gene ratio in both networks were the AGE-RAGE signaling pathway in diabetic complications, TNF signaling pathway, relaxin signaling pathway, IL-17 signaling pathway, and focal adhesion. Additional pathways, such as neuroactive ligand-receptor interaction, chemokine signaling pathway, and AMPK signaling pathway, were enriched with HXMMT targets. Thus, HXMMT has more therapeutic targets shared by different active ingredients and more abundant gene functions than CXC, which may be two major reasons why HXMMT is more strongly recommended than CXC as an auxiliary treatment for new-onset VH secondary to PDR. However, the underlying mechanisms need to be further elucidated.

## Figures and Tables

**Table 1:** Components of the compound Xueshuantong capsule and Hexuemingmu tablet [6, 7].

	Herbs
Compound Xueshuantong capsule (CXC)	*Radix salviae (Danshen), Panax notoginseng (Sanqi), Hedysarum multijugum* Maxim*. (Huangqi), Figwort root (Xuanshen)*

Hexuemingmu tablet (HXMMT)	*Radix salviae (Danshen), Chuanxiong rhizoma (Chuanxiong), Radix paeoniae rubra (Chishao), Gentianae radix et rhizoma (Longdan), Scutellariae radix (Huangqin), Cassiae semen (Juemingzi), Chrysanthemi flos (Juhua), Ecliptae herba (Mohanlian), Equiseti hiemalis herba (Muzei), Pollen typhae (Puhuang), Crataegus pinnatifida* Bunge *(Shanzha), Prunellae spica (Xiakucao), Rehmanniae radix praeparata (Dihuang), Plantaginis semen (Cheqianzi), Leonuri fructus (Chongweizi), Fructus ligustri lucidi (Nvzhenzi), Curcumae radix (Yujin), Cortex moutan (Mudanpi), Angelicae sinensis radix (Danggui)*

The names in parentheses are the Chinese names of the components.

**Table 2:** Genes/pathways mentioned in the discussion and whether CXC/HXMMT regulates their functions.

Genes/pathways	Role in PDR	CXC regulates?	HXMMT regulates?
Bcl-2, Bcl-2-associated X, and caspase-3	Apoptosis and neuroprotective effects	Yes [14]	Unknown
VEGF, ICAM-1, ET-1, PEDF, and occludin	Erythrocyte aggregation, plasma viscosity, acellular vessel, and pericyte loss	Yes [20, 21]	Unknown
PPAR signaling pathway	Protective effects	Unknown	Unknown
YAP	Protecting retinal vascular endothelial cells	Yes [6]	Unknown
MMP9 and IGF-1	Regulating retinal capillary cell apoptosis/neovascularization	Unknown	Unknown
APOD	Neovascularization	Unknown	Unknown
PECAM1	Maintenance of human vascular endothelial barrier integrity	Unknown	Unknown
YWHAB	Performing specific functions in rod photoreceptors	Unknown	Unknown
RACK1	Neovascularization	Unknown	Unknown
PARP1	Inflammation, cell death, and retinal disease progression	Unknown	Unknown
AGE-RAGE signaling pathway	Neovascularization/neuroprotection	Unknown	Unknown
Circular RNA COL1A2/miR-29b/VEGF	Neovascularization	Unknown	Unknown
VEGF/PI3K-Akt signaling pathway	Neovascularization	Unknown	Unknown
TNF signaling pathway	Inflammation	Unknown	Unknown

PDR, proliferative diabetic retinopathy; CXC, compound Xueshuantong capsule; HXMMT, Hexuemingmu tablet.

## Data Availability

No data were used to support this study.

## Abbreviations

CXC: Compound Xueshuantong capsule
HXMMT: Hexuemingmu tablet
CPM: Chinese patent medicines
PDR: Proliferative diabetic retinopathy
VH: Vitreous hemorrhage
GO: Gene ontology
KEGG: Kyoto encyclopedia of genes and genomes
DR: Diabetic retinopathy
DM: Diabetes mellitus
mRNA: Messenger RNA
DEmRNA: Differentially expressed messenger RNA
I-T: Ingredient-target
PPI: Protein-protein interaction
KEGG-T: Kyoto encyclopedia of genes and genomes-target
GEO: Gene expression omnibus
TCMSP: Traditional Chinese medicine system pharmacology
OB: Oral bioavailability
DL: Drug-likeness
KOBAS: KEGG orthology based annotation system
BP: Biological process
CC: Cellular component
MF: Molecular function
STZ: Streptozotocin
VEGF: Vascular endothelial growth factor
MCP-1: Monocyte chemoattractant protein-1.
